# Why do surgeons continue to perform unnecessary surgery?

**DOI:** 10.1186/s13037-016-0117-6

**Published:** 2017-01-13

**Authors:** Philip F. Stahel, Todd F. VanderHeiden, Fernando J. Kim

**Affiliations:** 1 Department of Orthopaedics, and Department of Neurosurgery, University of Colorado School of Medicine, Denver Health Medical Center, 777 Bannock Street, Denver, CO 80204 USA; 2 Department of Neurosurgery, University of Colorado, School of Medicine, Denver Health Medical Center, Denver, CO 80204 USA; 3 Division of Urology, Department of Surgery, University of Colorado, School of Medicine, Denver Health Medical Center, Denver, CO 80204 USA

Patient safety in surgery has historically suffered from a lack of physician-driven initiatives aimed at recognizing, preventing and mitigating medical errors and surgical complications [1]. In spite of a multiplicity of global patient safety initiatives, mandatory safety protocols and the introduction of surgical safety checklists, we continue to fall short of protecting our patients from preventable harm [2–6]. This unrecognized problem has escalated so far that medical errors currently rank as the 3^rd^ leading cause of death in the United States [7, 8] (Table 1). Strikingly, in the 21^st^ century, we still have to come to terms with the absurd reality that it is significantly safer to board a commercial airplane, a spacecraft, or a nuclear submarine, than to be admitted to a U.S. hospital [9–14]. What can surgeons do to protect their patients from the hidden dangers of an imperfect health care system? The most intuitive solution is to avoid complications originating from surgical treatment that may not be indicated or beneficial for patients in the first place. In other words, avoiding unnecessary surgery could be considered the most pragmatic approach towards reducing preventable surgical complication rates.

**Table 1:** Leading causes of death in the United States^a^

1. Heart disease (~614 000 deaths per year)	
2. Cancer (~591 000 deaths per year)	
3. Medical errors (~440 000 deaths per year)	

^a^Source:

▪ http://www.cdc.gov/

▪ *Journal of Patient Safety* 2013, 9:122–8

What do we mean by unnecessary surgery? We define this as any surgical intervention that is either not needed, not indicated, or not in the patient’s best interest when weighed against other available options, including conservative measures [1, 15]. From a historic perspective, the threat of unnecessary surgery has been publicized as far back as the 1950s, when Dr. Paul Hawley, the Director of the American College of Surgeons (ACS), stated that *“the public would be shocked if it knew the amount of unnecessary surgery performed (…)”* [16]. More than twenty years later, in 1976, the American Medical Association (AMA) called for a congressional hearing on unnecessary surgery, claiming that there were *“2.4 million unnecessary operations performed on Americans at a cost of $3.9 billion and that 11,900 patients had died from unneeded operations (…)”* [17].

In 2016, the existence of unnecessary surgery remains a daunting reality that continues to expose our patients to an unjustified surgical risk [18]. For example, multiple clinical trials have shown that spinal fusions for back pain do not lead to improved long-term patient outcomes when compared to non-operative treatment modalities, including physical therapy and core strengthening exercises [19, 20]. In spite of these insights from high-quality trials, spinal fusion rates continue to dramatically increase in the United States [18]. Another relevant example is arthroscopic partial meniscectomy, one of the most commonly performed surgical procedures in the world [21]. This minimally invasive surgery allows treating internal knee damage through small percutaneous skin incisions, with a fast-track postoperative recovery period. In the United States alone, surgeons perform approximately 700,000 arthroscopic partial meniscectomies every year. Strikingly, a recently published prospective randomized controlled trial (“Finnish Degenerative Meniscal Lesion Study”/FIDELITY trial) that assessed patient outcomes after arthroscopic meniscal trimming compared to sham surgery revealed *no* benefit for patients from the routine surgical procedure at 12 months follow-up [22]. Actually, considering the risk for patients sustaining a severe intra- or postoperative complication, no surgical procedure should be considered “routine” from the patient’s perspective [23]. Yet, until present, a change in practice has not occurred, and arthroscopic meniscectomies continue to be performed on hundreds of thousands of patients in the United States every year [24, 25].

Consider this provocative analogy: If surgery were a pharmaceutical drug, the procedure would be required to undergo scrutiny of testing its safety and feasibility in phase 1 and 2 trials. Subsequently, its efficacy would have to be proven in prospective randomized controlled trials prior to approval by the Food and Drug Administration (FDA) [18]. Yet, the FDA does *not* regulate surgical procedures. Common sense would impose the expectation that whenever new level 1 evidence disproves a benefit for a certain surgical procedure, the ineffective practice would be called into question and abandoned immediately. This is obviously not the case in the field of surgery.

The title of this editorial asks, “Why do surgeons continue to perform unnecessary surgery?” To phrase it another way, one might pose the question, “Why would a reasonable surgeon consider performing unneeded surgical procedures?” From a surgeon’s perspective, two distinct answers appear intuitive:

1. We perform surgery because we have been trained to do so and because “we have always done it this way” or we simply do not know any better. In German psychology, this behavior is analogous to a historic entity termed “Funktionslust” [1].
2. We are incentivized to perform surgical procedures, either for financial gain, renown, or both.

As representatives of the most privileged and rewarding profession on Earth, it is our duty as surgeons to be unwavering patient safety advocates. This mandates that we recognize the common - yet extremely dangerous - incentives of unnecessary surgery and their potentially deleterious effects on our patients. Once these “hidden threats” are recognized and mitigated, surgeons can begin to foster a transparent culture of shared decision-making and thereby form a true partnership with their patients [26]. Under this evolving paradigm, patients are encouraged to participate in the choice of their treatment based on the best available scientific evidence, while surgeons take into consideration and respect their patients’ personal values, fears, and expectations [26]. By embracing patient safety as a core responsibility for surgeons, we have the opportunity of eliminating the “phantom menace” of unnecessary surgery and the associated risk of preventable patient harm.

This responsibility is not negotiable. The onus is on us.
